# Beyond the Transparent Barrier: A Domain Visualization and Integrative Review of Contemporary Research on Gender-Based Professional Stasis

**DOI:** 10.12688/f1000research.180295.1

**Published:** 2026-05-29

**Authors:** Pankaj Kumar Tyagi, Praveen Kumar, Asokan Vasudevan, Premendra Kumar Singh, CS Jyoti Arora, Trinkul Kalita, Purvi Pujari

**Affiliations:** 1 University Institute of Tourism and Hospitality Management, Chandigarh University, Sahibzada Ajit Singh Nagar, Punjab, India; 2 School of Hotel and Tourism Management, VIT University, Vellore, Tamil Nadu, India; 3 Faculty of Business and Communications, INTI International University, Negeri Sembilan, Malaysia; 4 Centre for Distance and Online Education, Sharda University, Greater Noida, Uttar Pradesh, India; 5 School of Management Studies, CGC University, Mohali, Punjab, India; 6 Faculty of Commerce and Management, Assam Down Town University, Guwahati, Assam, India; 7 Vijay Patil School of Management, DY Patil University Deemed to be University, Navi Mumbai, Maharashtra, India

**Keywords:** Glass ceiling; Knowledge domain visualization; Thematic integration; Women in leadership; Gender inequality; Systematic literature review; PRISMA; Career barriers; Leadership development; ADO framework; Gendered inequality; Quality education

## Abstract

**Background:**

Despite decades of policy and reforms, diversity initiatives and greater awareness about the importance of gender equality, women still remain notably absent from leadership roles in most of the countries around the world. The persistence of the glass ceiling highlights the need for a deeper understanding of the intellectual evolution and structural drivers of gendered career barriers.

**Methods:**

A PRISMA-guided systematic literature review approach, combining knowledge domain visualization with thematic integration was used to track the glass ceiling research. Bibliographic coupling, co-citation analysis and keyword co-occurrence mapping using VOSviewer were performed which helped in identifying most influential authors, documents, countries and publication sources.

**Results:**

Analysis shows four interrelated thematic areas of contemporary glass ceiling research: (1) Gender Inequality in Academic and Labor Markets— How Institutions Perpetuate the Divide; (2) Breaking Through: Organizational Mechanisms & Career Pathways; (3) The Human Factor: Leadership Development & Career Identity; (4) Unlocking Potential: Diversity Initiatives & Empowerment Strategies. Guided by these insights, the study develops an integrative conceptual framework from the Antecedents–Decisions–Outcomes (ADO) perspective where structural and institutional conditions act as antecedents that frame organizational/societal and individual career decisions leading to outcomes relevant to careers such as leadership representation, advancements opportunities or gender equity in leadership positions.

**Conclusion:**

This study presented a systematic integration of knowledge domain along with suggesting an ADO-based conceptual framework to provide innovative synthesis of the transdisciplinary scholarly work in contemporary glass ceiling research. These findings offer actionable lessons for policymakers, organizations and diversity practitioners who seek to implement effective interventions that will contribute to reducing systemic gender inequalities in leadership.

## 1. Introduction

Over the last four decades, research on the glass ceiling has dramatically increased as concern for women’s under-representation in top leadership has continued following a sharp rise in their educational qualifications and labour market participation rates. First introduced as a metaphor characterizing the “invisible, artificial barriers which block women from getting promotions,” the glass ceiling has become one of its most prominent metaphors to understand how gendered norms, organizational practices and policy failures intersect in shaping women’s career paths ( Mohamed et al., 2023; Çitil, 2022). Cumulative empirical evidence documenting persistent vertical segregation has accrued across sectors and regions, with scholars now asking not just if a glass ceiling exists, but also how it is conceptualized, where it is most salient, and how research on the topic itself has developed over time ( Maheshwari & Lenka, 2022; Purcell et al., 2010; Jackson & O’Callaghan, 2009). In parallel, the volume of academic work on the glass ceiling has grown to the point that systematic mapping of the field has become both possible and necessary. Beyond counting papers and citations, scholars have begun to interrogate the **conceptual and thematic structure** of glass ceiling research. Co-word and network analyses show that work on the glass ceiling is tightly interwoven with broader metaphors of gendered inequality (e.g., glass cliff, sticky floor, leaky pipeline) and with themes such as leadership, organizational culture, discrimination, and work–family conflict ( Martínez-Fierro & Sancho, 2021; Maheshwari & Lenka, 2022; Grangeiro et al., 2021a). Meanwhile, reviews also point to crucial gaps such as little engagement with intersectionality, sectoral blind spots and differential focus on policy instruments and organizational interventions ( Maheshwari & Lenka, 2022; Grangeiro et al., 2021b). By adopting an integrated bibliometric and thematic approach, it seeks to:

(1) *identify intellectual structures through bibliographic coupling, co-citation analysis, and keyword co-occurrence analysis in glass ceiling literature;*
(2) *highlight underexplored areas and propose future research directions in the area of glass ceiling research.*

## 2. Methodology

**Figure 1.**
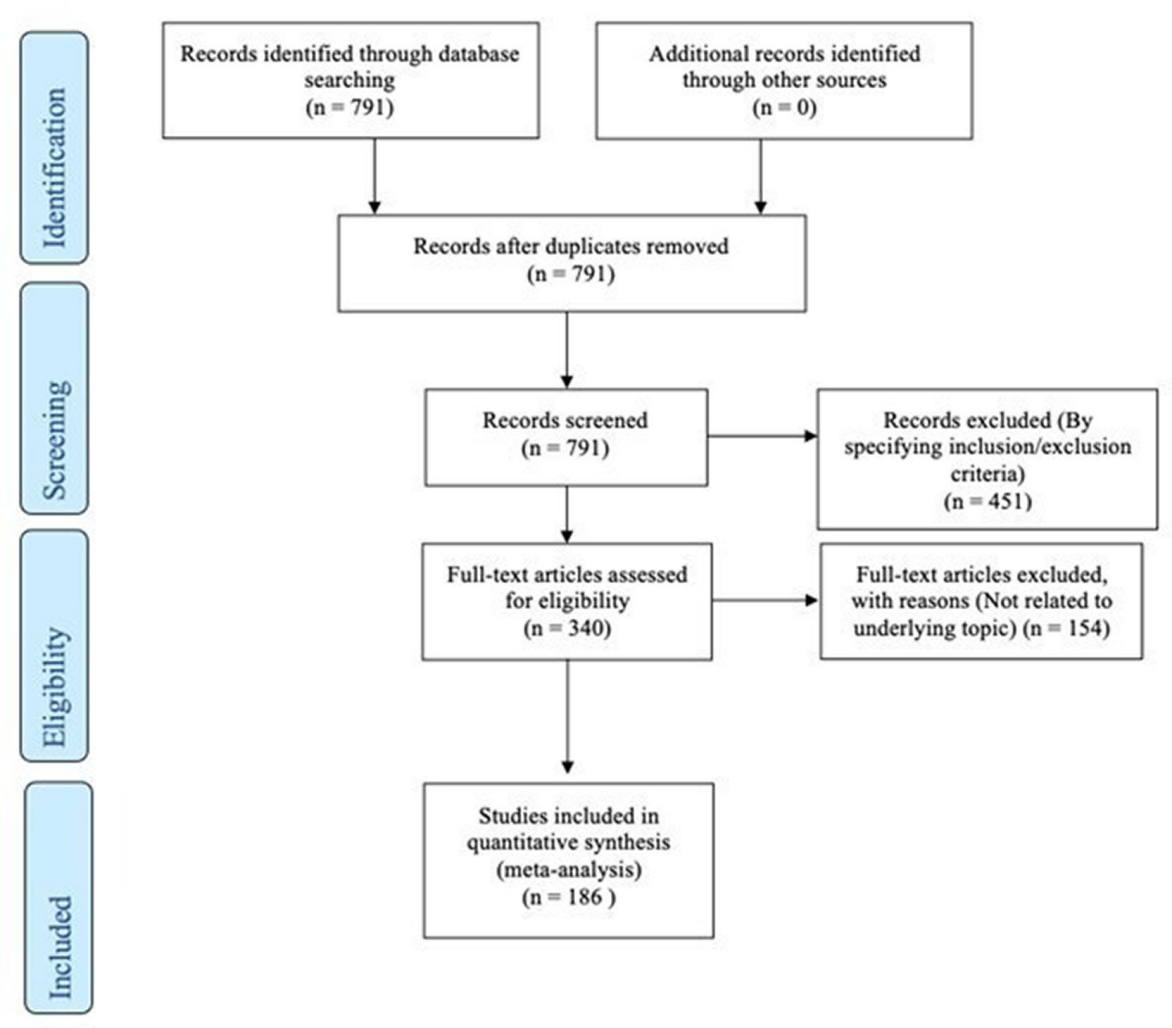
Methodology for data collection following PRISMA guidelines.

This study utilizes a systematic three-phase investigation consisting of PRISMA based review, bibliometric and thematic analysis to examine the glass ceiling literature. The analysis was performed on peer-reviewed journal articles in English language published between 2015–2025 retrieved from Scopus, containing the keywords glass ceiling, women’s career advancement, career progression, women in leadership and gender based barriers. They focused explicitly on identified barriers to women’s careers in the domains of social sciences, business, economics, humanities and psychology and excluded non-peer-reviewed works as well as studies published in any language other than English or that were marginally relevant. In total, after PRISMA screening the number of records was limited to a final set of 186 high-quality studies retaining methodological rigour and relevancy ( Figure 1). This approach increases the reliability of the findings and generates an overall mapping of the intellectual architecture of research on glass ceiling. Bibliometric analysis was conducted to assess research output, collaboration patterns, and intellectual links through bibliographic coupling and co-citation analysis among the selected papers. VOSviewer visualizations and keyword co-occurrence, using thematic analysis to determine dominant topics and their linkages.

## 3. Findings

This section presents the results of bibliometric and thematic analysis.

### 3.1 Bibliometric findings

**3.1.1 Bibliographic coupling of countries**

**Figure 2.**
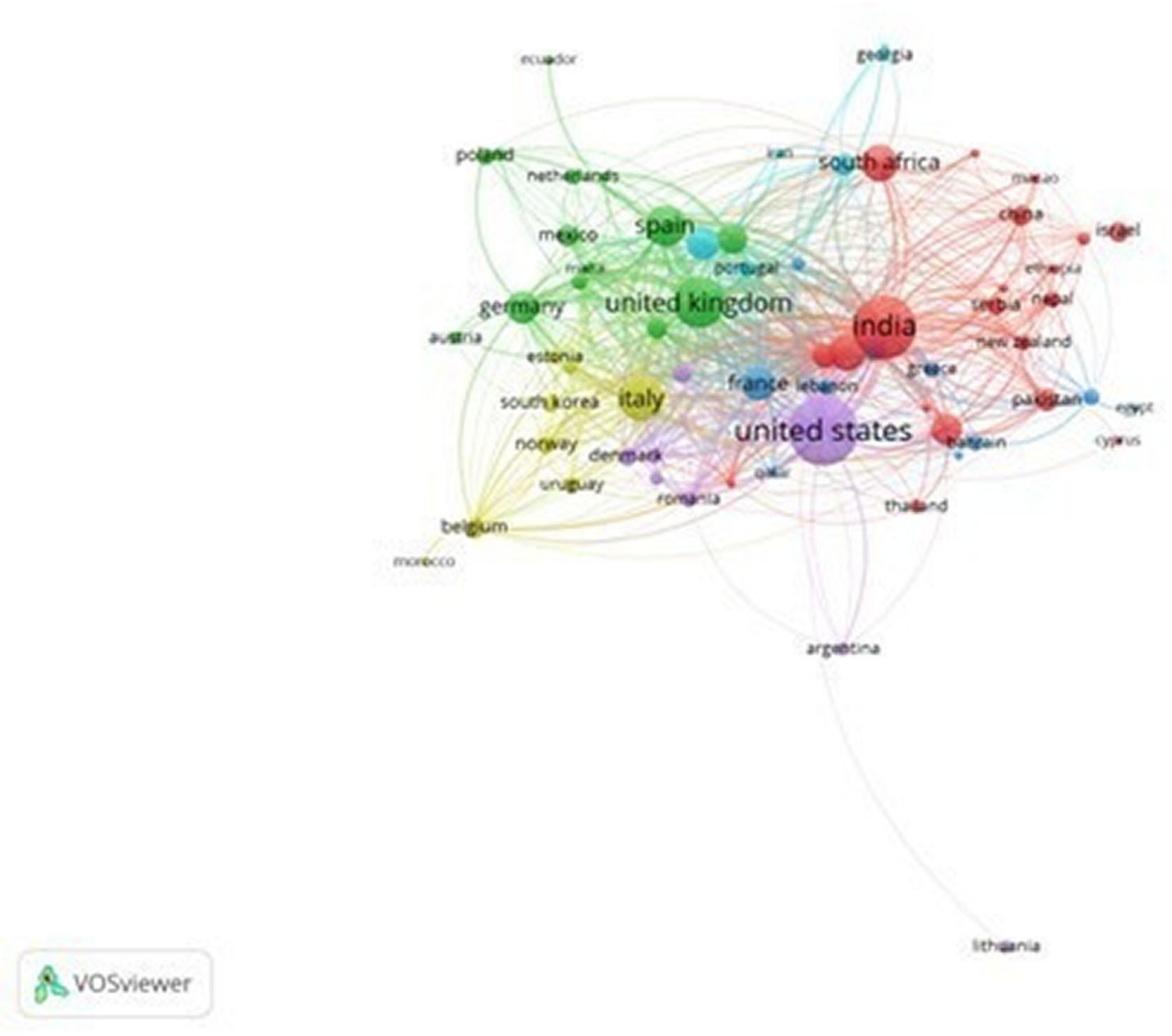
Bibliographic coupling of countries (Source: VOSviewer).

Bibliographic coupling of countries provides an overview of the global glass ceiling literature ( Figure 2). Most economically advanced countries (USA, UK, India, China, Germany, Spain and Italy) are densely packed clusters with tightly connected collaboration networks. Peripheral countries show less contribution in crossing the glass ceiling of academia, whereas those belonging to Europe (green cluster) and Asia (red cluster) suggest that strong regional research coherence exists. A striking lack of papers from Africa and Latin America suggests that the literature dealing with gender-based barriers to careers is primarily shaped by developed and developing economies.

**3.1.2 Bibliographic coupling of documents**

**Figure 3.**
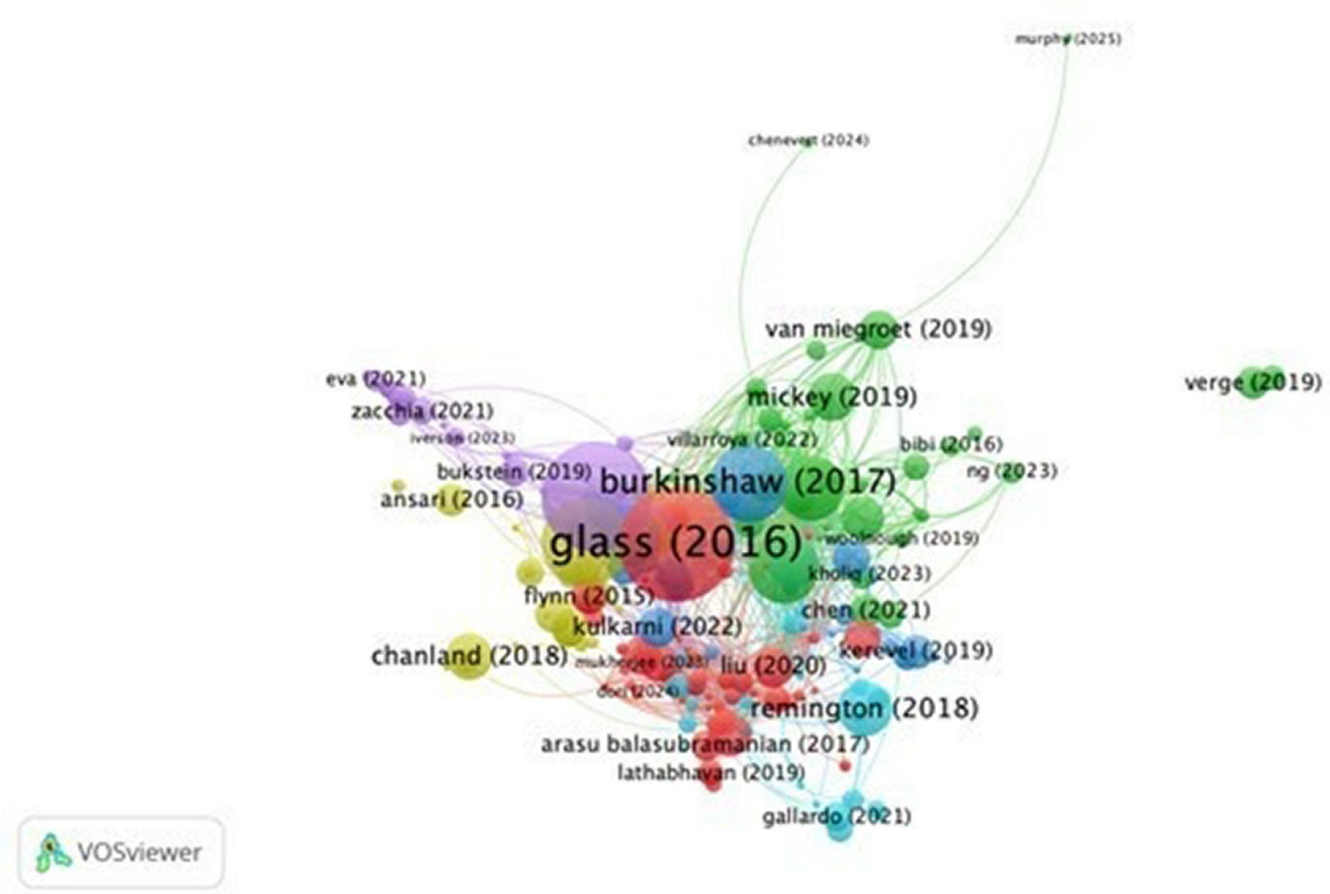
Bibliographic coupling of documents (Source: VOSviewer).

The bibliographic coupling of documents analyzes the cognitive structure and temporal distribution of glass ceiling literature ( Figure 3). The segment shows that there’s a spreading of topics: yellow nodes are focused on the barriers in organisations, red on gender dynamics and green addresses new challenges such as intersectionality and digital transformation. The temporal analysis of 2015–2025 shows a fast pace research momentum and it is more vigorous post-2019, which indicates an augmented scholarly curiosity toward gender bias barriers to a career.

**3.1.3 Bibliographic coupling of sources**

**Figure 4.**
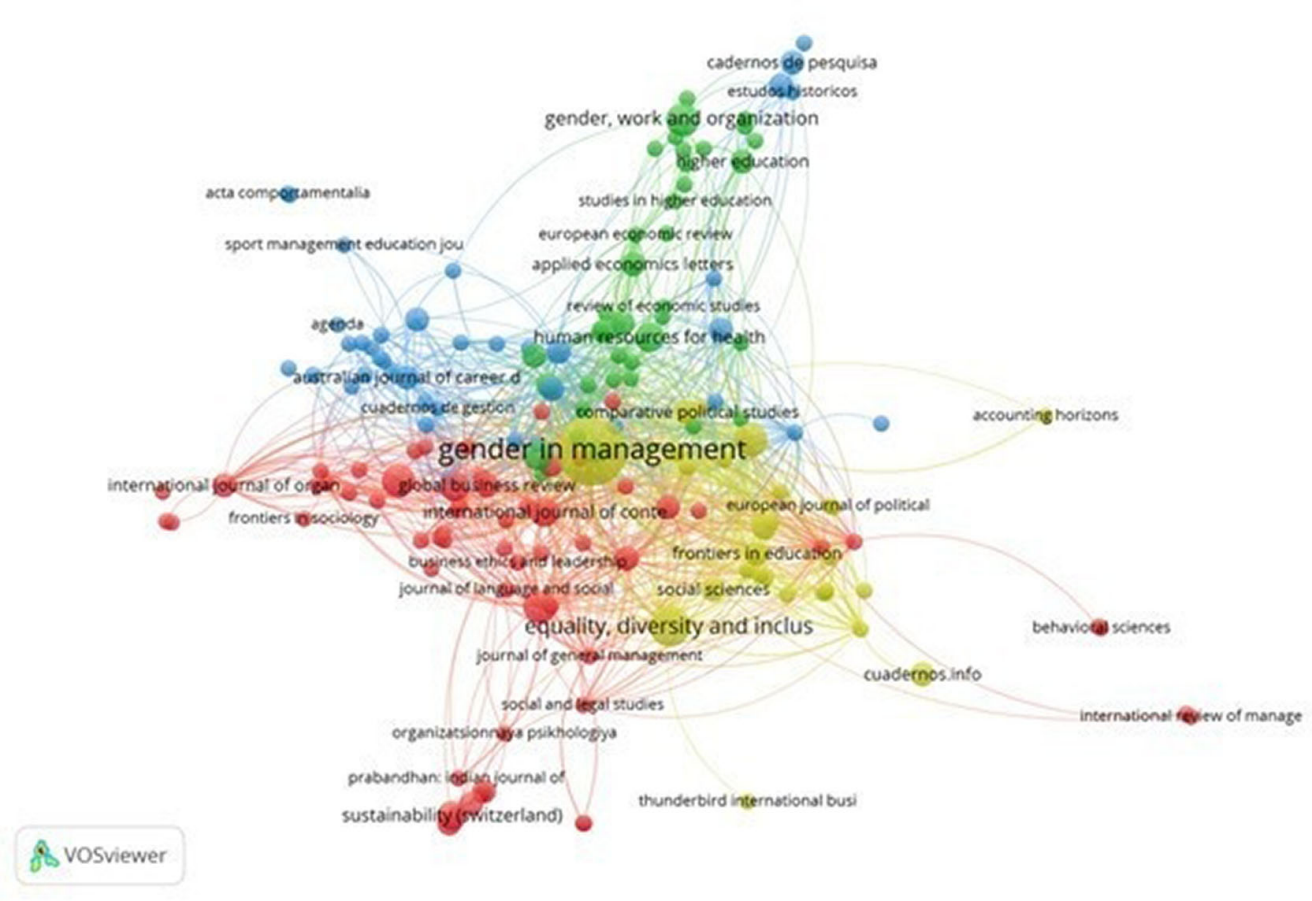
Bibliographic coupling of documents (Source: VOSviewer).

Bibliographic Coupling of Sources ( Figure 4) maps the disciplinary landscape and journal networks, which have shaped glass ceiling research. It illustrates three interconnected publication ecosystems: Management and organisational studies (red cluster) includes the field’s core, gender-centred journals like ‘Gender in Management’ and ‘Business Ethics & Leadership’; higher education an institutional studies (green cluster), are anchored around the journal’Studies in Higher Education’ as well as’European Economic Review; social sciences/interdisciplinary outlets encompassing ‘Frontiers in Sociology’ or ‘International Journal of Organisational Psychology’. They also signal how glass ceiling work has been framed almost exclusively in organizational and management terms, possibly at the expense of a more sociological or psychological analysis with an intersectional element.

**3.1.4 Co-citation analysis of authors**

**Figure 5.**
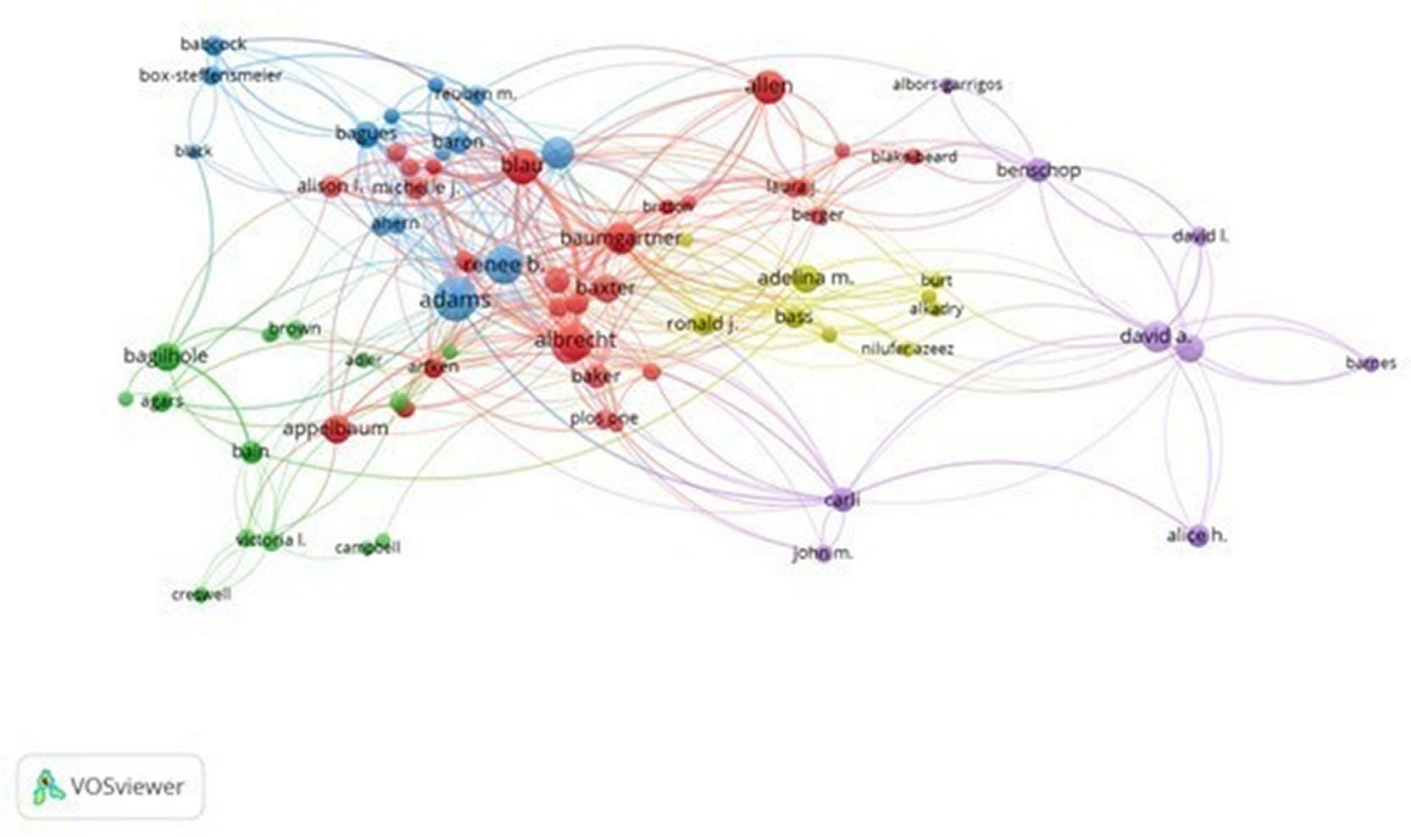
Co-citation analysis of authors (Source: VOSviewer).

Author co-citation analysis portrays the intellectual architecture and roots of glass ceiling literature ( Figure 5). The tightly knitted red cluster highlights the authors in organizational behaviour and management who largely dominate citation behaviors; the blue cluster is made up of scholars whose works mainly focus on institutions and education, and further outside in green/purple are emerging or relatively specialized contributors. For example, there is high co-citation rates among authors Allen, Baxter, Albrecht, and Baumgartner, as reflected in the red cluster.

**3.1.5 Co-citation analysis of sources**

**Figure 6.**
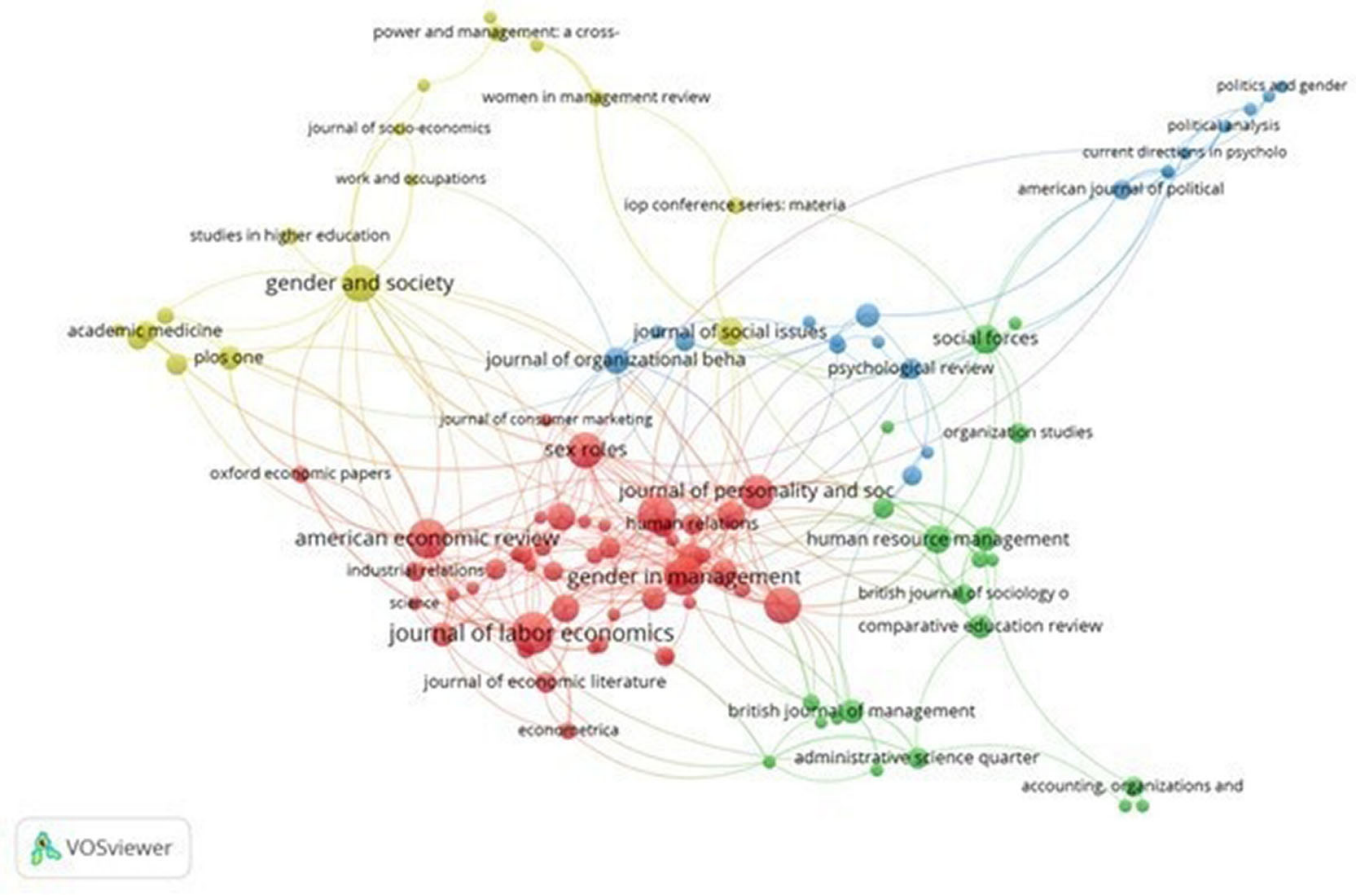
Co-citation analysis of sources (Source: VOSviewer).

Co-citation analysis of sources reveals an increasing disciplinary fragmentation and the thematic priorities that define glass ceiling discourse, with three different publication ecosystems: management and organizational studies (red cluster) revolving around “Gender in Management” and “Journal of Labor Economics” stressing structural barriers/workplace dynamics; social sciences/psychological theory (blue cluster) based on “Psychological Review” and “Journal of Social Issues” facilities personal, psychological dimensions: higher education/gender studies (yellow/green cluster) involving “Studies in Higher Education”, “Gender & Society” dealing with institutional/societal contexts ( Figure 6). The overwhelming presence of economic and management literature indicates that research on the glass ceiling is more concerned with labour market analysis and organizational fixes rather than more general sociological or intersectional hierarchization. Weak links between clusters suggest that management-themed literature exists fairly independently from gender and psychology texts and there is little theoretical cross-fertilization, failing to account for potential psychological, cultural, and system intersections.

### 3.2 Thematic analysis

**Figure 7.**
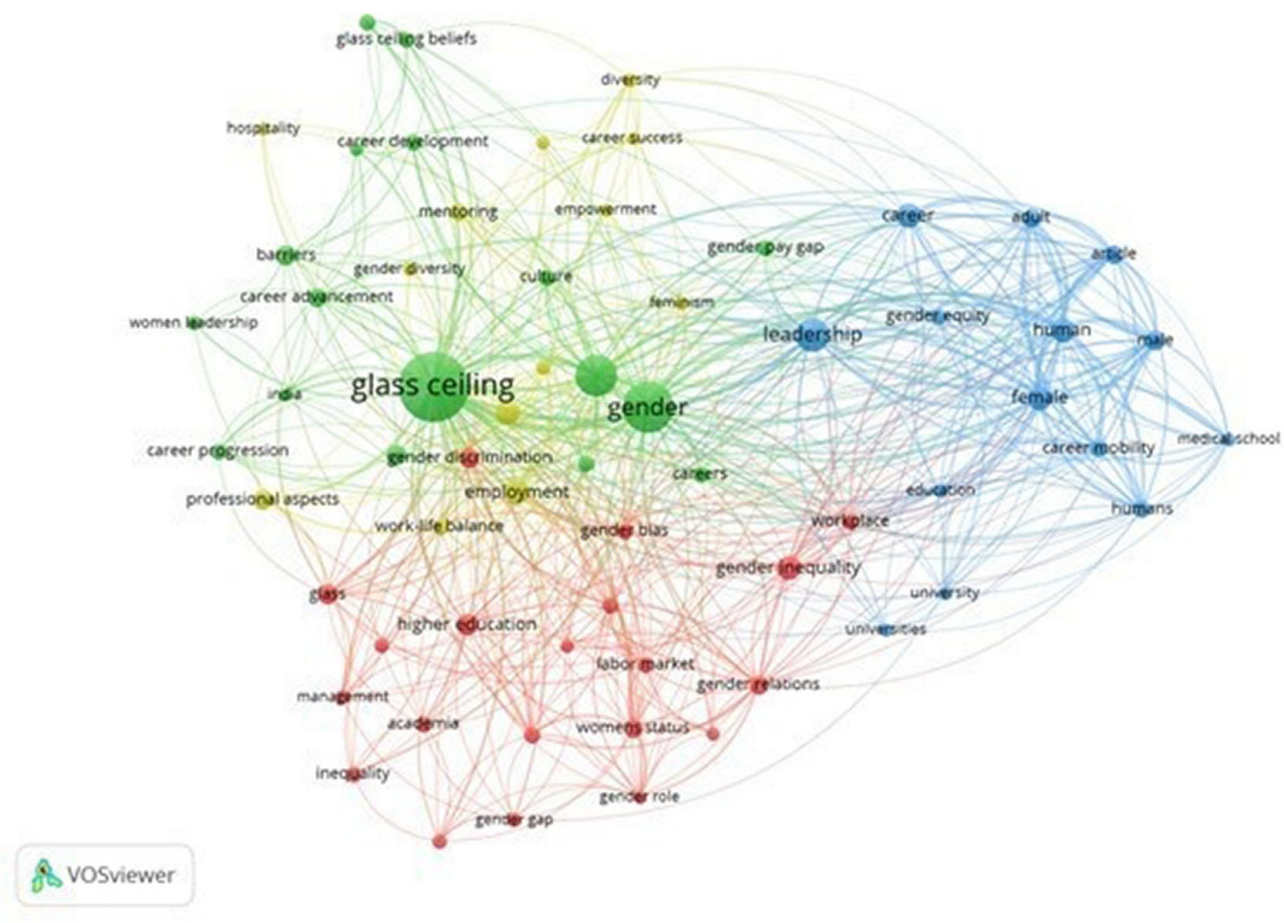
Keywords co-occurrence analysis (Source: VOSviewer).

In this section, the authors have done thematic analysis based on the themes identified using keywords co-occurrence analysis ( Figure 7). The following four emerging themes were identified: (1) Gender Inequality in Academic and Labor Markets— How Institutions Sustain the Divide; (2) Breaking Through: Organizational Mechanisms & Career Pathways; (3) The Human Factor: Leadership Development & Career Identity; (4) Unlocking Potential: Diversity Initiatives & Empowerment Strategies.

**3.2.1 Gender inequality in academic & labor markets: How institutions sustain the divide (Red Cluster)**

Gender inequality in academia and labor markets is not merely a story of individual choices but rather the product of structural and cultural arrangements that funnel women into lower-status positions with weaker returns on education. Research across disciplines and countries indicates persistent gaps in representation, pay, security and authority, even as women’s educational attainment has surged. Women are still underrepresented in senior academic roles and top institutional leadership, even where they currently make up the majority of graduates ( Rosa & Clavero, 2021; O’Connor, 2020; Winslow & Davis, 2016; Wieczorek-Szymańska, 2020; Alshdiefat et al., 2024; Yousaf & Schmiede, 2017). Research suggests that fewer women are in top ranks, worse pay and promotion prospects, more precarious contracts and heavier teaching or administration loads ( Eslen-Ziya & Yildirim, 2021; Vohlídalová, 2021; Winslow & Davis, 2016; Nkosi & Maphalala, 2025; O’Keefe & Courtois, 2019; Yousaf & Schmiede, 2017). Furthermore, women concentrated in fields with weaker labor-market returns (humanities, care professions), underrepresented in high-pay STEM and tightly job-linked majors ( Tan et al., 2025; Zheng & Weeden, 2023; Barone & Assirelli, 2019; Fontanella et al., 2019). These patterns translate into lower wages, slower advancement, and “maternity fees” in labor markets ( Tan et al., 2025; Fontanella et al., 2019). Academic organizations are described as **“gendered and gendering”:** norms of merit and excellence are coded masculine, privileging uninterrupted linear careers, presenteeism, and male networks ( Rosa & Clavero, 2021; Khan et al., 2024; O’Connor, 2020; Winslow & Davis, 2016; O’Keefe & Courtois, 2019; Yousaf & Schmiede, 2017). Strong hierarchies correlate with women’s perceptions that gender harms their prospects and limits future advancement ( Eslen-Ziya & Yildirim, 2021; Khan et al., 2024; O’Keefe & Courtois, 2019). The informal exclusion from networks, mentorship and reputational opportunities reinforces formal inequalities ( Eslen-Ziya & Yildirim, 2021; Winslow & Davis, 2016; O’Keefe & Courtois, 2019; Yousaf & Schmiede, 2017; ( O’Keefe and Courtois 2019). Gender intersects with class, race/ethnicity, religion, and national context; disadvantages compound for marginalized women and in conservative or post-colonial settings ( Rosa & Clavero, 2021; Khan et al., 2024; Fontanella et al., 2019; Crimmins et al., 2023; Alshdiefat et al., 2024). Academic capitalism and competitive funding can create new opportunities for a subset of women, while exacerbating inequalities among women by class and partnership status ( Gaiaschi 2025). Everyday sexism and local power relations persist to impede genuine inclusion even in institutions rewarded for their plans for gender equality ( Rosa & Clavero, 2021; O’Connor, 2020; Crimmins et al., 2023) have limited transformative effect if core structures and cultures are protected through certain forms of covering or adaptation ( Rosa & Clavero, 2021; Khan et al., 2024; O’Connor, 2020; Crimmins et al., 2023; Wieczorek-Szymańska, 2020; Nkosi & Maphalala, 2025; Ceci et al., 2023; Winslow & Davis, 2016; Nkosi & Maphalala, 2025; O’Keefe & Courtois, 2019). Systems-wide solutions involve going beyond individual stigmas through improved policies for work-life and caring responsibilities, equity-sensitive funding mechanisms, transparent career advancement criteria with robust mentorship as institutional transformation not simply add-ons ( Rosa & Clavero, 2021; Khan et al., 2024; O’Connor, 2020; Wieczorek-Szymańska, 2020; Nkosi & Maphalala, 2025; Alshdiefat et al., 2024). Educational advances have not brought about equivalent status, pay or security, especially at the top and in high-return fields. Reforms that merely “fix women” aren’t enough; the research converges with others, including my own, concluding that institutional structures, career logics and cultural norms must be remade if the academic and labor market achievements of women are ever to produce genuinely equal returns.

**3.2.2 Breaking through: Organizational mechanisms & career pathways (Green Cluster)**

In every sector and country, women’s career advancement is limited by organizational cultures, structures, and career systems that are premised on gendered expectations and bias. Unseen “glass ceiling” hurdles arise throughout banking, universities, health, ICT, policing, construction and more corporate environments, even when women are highly qualified ( Lyan et al., 2025; Ganiyu et al., 2018; Shrestha et al., 2023; Hirayama & Fernando, 2018; Tešanović et al., 2024; Khan & Khan, 2022; Shrestha, 2025; Naz et al., 2025; Kumari & Kaur, 2025; Khan et al., 2024). Corporate cultures and practices in finance and banking exert a strong, adverse influence on women’s chances of promotion, accounting for over fifty per cent of the variation in career advancement ( Shrestha et al., 2023; Shrestha, 2025). Senior positions and visibility are limited by biased promotion processes, gendered stereotypes like “think manager–think male”, and male-dominant cultures of leadership ( Lyan et al., 2025; Thelma & Ngulube, 2024; James, 2025; Ganiyu et al., 2018; Naseviciute & Jucevičienė, 2023; Hirayama & Fernando, 2018; Saville et al., 2024; Thakurathi, 2025). One potential explanation of this glass ceiling phenomenon limiting career success encompasses the role that work–family conflict contributes; in other words, higher conflict predicts a decrease in accessing opportunities to achieve ( Hirayama & Fernando, 2018; Khan & Khan, 2022; Todak et al., 2025). Motherhood attracts closer scrutiny, slower advancement and altered relationships at work, although the effects differ by context and support structures ( Torres et al., 2024). Long hours, highly structured career ladders, and masculine cultures make work–life balance challenging and discourage leadership aspirations in surgery ( Francis, 2017), policing, construction, ICT and health ( Naseviciute & Jucevičienė, 2023; Hirayama & Fernando, 2018; Saville et al., 2024; Todak et al., 2025; Petelin et al., 2025). Access to mentors, sponsors and influential networks is a chronic bottleneck; where mentoring exists, it enhances career progression and retention, particularly in male-dominated sectors ( Lyan et al., 2025; Francis, 2017; Thelma & Ngulube, 2024; Naseviciute & Jucevičienė, 2023; Saville et al., 2024; Thakurathi, 2025; Petelin et al., 2025). Some sectors (e.g., construction, ICT) reveal that individual agency, continuing to build skills—strategic networking and self-confidence—can help women navigate “contest” career systems but will not remove structural bias ( Francis, 2017; Naseviciute & Jucevičienė, 2023; Petelin et al., 2025). Interventions with evidence include: flexible and diverse career paths, family-friendly policies, transparent criteria for promotion, gender-transformative leadership development and robust anti-harassment systems ( Lyan et al., 2025; Shrestha et al., 2023; Hirayama & Fernando, 2018; Torres et al., 2024; Saville et al., 2024; Naz et al., 2025; Kumari & Kaur, 2025). Glass ceilings stubbornly remain due to interdependent individual, organizational and structural barriers, but clear evidence shows that we can advance women’s career progression via inclusive cultures, flexible career systems and access to mentoring—not the individual fixes that are often more visible ( Lyan et al., 2025; Naseviciute & Jucevičienė, 2023; Torres et al., 2024; Saville et al., 2024; Kumari & Kaur, 2025; Khan et al., 2024).

**3.2.3 The human factor: Leadership development & career identity (Blue Cluster)**

Research studying the “human factor” in women’s careers highlights how capabilities, mindsets and identities developed early in life interplay with opportunities and constraints over time. Agency is important, but it is never exercised outside social, or organizational and cultural systems. College and early adulthood are key developmental periods as achievement orientations and leadership orientation in college predict attainment of senior leader roles and higher pay nearly 30 years later ( Offermann et al., 2020). For young women and girls, leader identity is influenced by social connections, personal attributes, meaningful involvement and social identities e.g. gender or race ( Dixon et al., 2023). Leadership-only programs for women show that identity work involves validation, new narratives of belonging, and learning there are **multiple legitimate ways to lead**, not just “leading like a man” ( Brue & Brue, 2018). Authentic and women-only leadership programs increase **self-efficacy, agency, and ownership** of career moves through reflection, life-story work, and relational authenticity ( Martínez-Martínez et al., 2021; Lee et al., 2024; Brue & Brue, 2018; Round et al., 2024).

Some women physicians and scientists develop their own paths to leadership, participating in early-career development programs that align with their values around the goals of leading, negotiating, articulation of vision and alignment between leadership with well-being and family roles ( Lee et al., 2024). Leadership emergences with strong leadership identity increased greatly when self-efficacy is formed as part of psychological capital through programs that take it as direct target ( Deroncele-Acosta et al., 2025; Round et al., 2024) As many young women embark on their work lives, they approach the pursuit of high-status jobs with optimism and ambition but soon find their ambition falls ( Beaupre, 2022), hindered by a lack of satisfaction with cultures of leadership at some levels and by expected costs to their personal life. Higher psychological capital such as optimism, hope, resilience, self-efficacy, along with intrinsic motivation, acts as a protective factor for mental health and performance among women doctoral students ( Deroncele-Acosta et al., 2025; Round et al., 2024). Across contexts (Nepal, Vietnam, Chile), education, continuous learning, performance, and family/mentor support underpin women’s **career mobility and persistence** in demanding fields ( Gómez-Arízaga et al., 2025; Thaiduong, 2025; Adhikary & Shrestha, 2023; Damaske & Frech, 2016). Women’s leadership trajectories are strongly shaped by early leader identity, accumulated human capital, and psychological resources like self-efficacy and resilience, all reinforced by developmental relationships.

**3.2.4 Unlocking potential: Diversity initiatives & empowerment strategies (Yellow Cluster)**

Research on “unlocking potential” centers on how concrete policies, programs, and beliefs can either dismantle or quietly reinforce the glass ceiling. Reviews stress that **unconscious bias,** glass ceiling beliefs, and masculine leadership norms continue to shape recruitment, promotion, and culture, so one-off diversity actions are insufficient ( Kumari & Kaur, 2025; Thakur, 2025; Thelma & Ngulube, 2024; Galsanjigmed & Sekiguchi, 2023; Lyan et al., 2025). Effective strategies must go beyond token female board quotas toward **deep cultural change**, transparent promotion, and strict anti-discrimination enforcement ( Kumari & Kaur, 2025; Thakur, 2025; Lyan et al., 2025). Formal, women-focused mentoring is repeatedly identified as a **core empowerment tool**, building self-advocacy, access to stretch roles, and a pipeline of female talent ( Thelma & Ngulube, 2024; Groves, 2021; Rampersad, 2024; Dashper, 2020; Mathetha & Dhanpat, 2025). In hospitality and academia, long-term mentoring programs both support individual careers and begin to **challenge gendered definitions of success and leadership** ( Rampersad, 2024; Dashper, 2020). Senior women are “empowerment multipliers”, and inclusive HR programs such as fair staffing, mentoring, pay equity, flexible work and leadership development positively impact on the career mobility of women when they are systematically implemented ( Mathetha & Dhanpat, 2025; Kumari & Kaur, 2025; Aggarwal, 2025 James, 2025). In all sectors, successful empowerment combines changes of structures with human-capital initiatives, and strong impact derives from accountability and deep cultural change rather than symbolic exercises in diversity ( Hamzaoui et al., 2025; Al-Naqbi & Aderibigbe, 2024; Mehmood, 2025; Maheshwari & Nayak, 2020).

## 4. Conceptual framework

**Figure 8.**
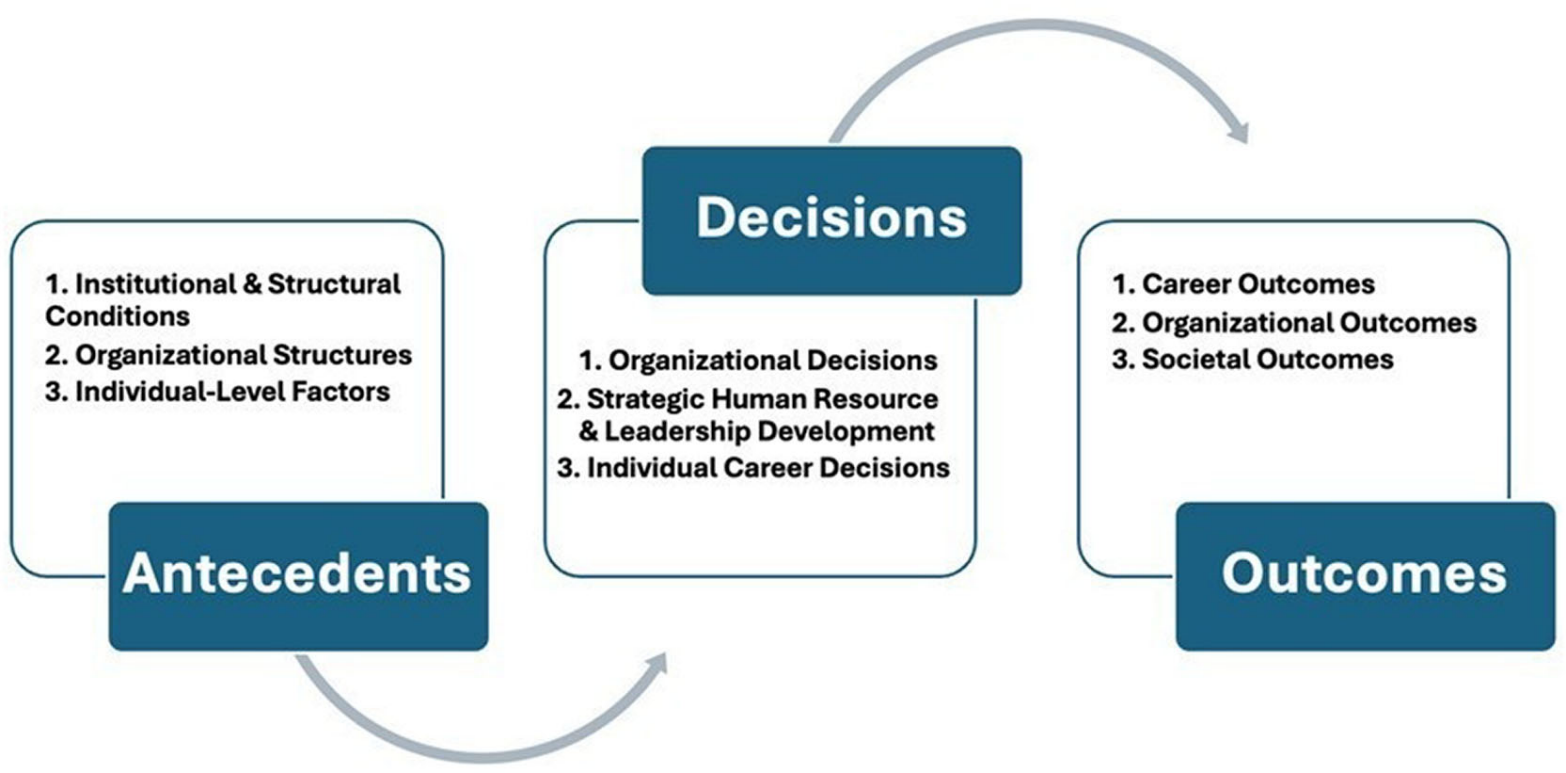
ADO framework for understanding the glass ceiling in leadership.

This study proposes an integrative Antecedents–Decisions–Outcomes (ADO) framework to systematically explain the multi-level drivers of the glass ceiling, linking structural and institutional conditions (antecedents) with organizational and individual career choices (decisions) to their resultant leadership and equity outcomes ( Table 1 and Figure 8).

**Table 1.** ADO framework for understanding the glass ceiling in leadership.

ADO component	Dimensions	Key elements	Illustrative insights from literature
**Antecedents**	**Institutional & structural conditions**	Gender norms, societal expectations, labor market segmentation, policy gaps	Persistent gender norms and institutional biases continue to shape unequal access to leadership roles, reinforcing systemic barriers ( Eagly & Karau, 2002; Ridgeway, 2011; Heilman, 2012; World Economic Forum, 2023; UN Women, 2022).
		Regulatory frameworks and diversity mandates	Weak enforcement of gender equality policies limits their effectiveness in addressing leadership disparities ( Terjesen et al., 2016; Post & Byron, 2015; OECD, 2020).
	**Organizational structures**	Gendered organizational cultures, glass ceiling effects, lack of mentorship	Organizational hierarchies and informal networks perpetuate exclusion from leadership pipelines ( Cotter et al., 2001; Ibarra et al., 2013; Ely et al., 2011; Catalyst, 2020).
		Pay gaps and promotion biases	Women face slower career progression due to implicit bias and unequal evaluation standards ( Blau & Kahn, 2017; Castilla & Benard, 2010; Correll et al., 2007).
	**Individual-level factors**	Career aspirations, self-efficacy, identity constraints	Gendered socialization affects leadership ambition and confidence levels ( Bandura, 1997; Hoyt & Murphy, 2016; Sandberg, 2013).
		Work–life balance pressures	Care responsibilities disproportionately affect women’s career continuity ( Hochschild & Machung, 2012; Goldin, 2014; Bianchi & Milkie, 2010).
**Decisions**	**Organizational decisions**	Hiring, promotion, leadership selection criteria	Organizational decision-making often reflects unconscious bias, limiting women’s advancement ( Rivera, 2017; Bohnet, 2016; Kalev et al., 2006).
		Diversity policies and inclusion strategies	Firms adopting structured diversity programs show improved gender representation ( Dobbin & Kalev, 2016; Nishii, 2013; Shore et al., 2011).
	**Strategic human resource & leadership development**	Mentorship, sponsorship, training programs	Leadership development initiatives enhance women’s advancement opportunities ( Ibarra et al., 2010; Ragins & Kram, 2007; Allen et al., 2004).
		Flexible work arrangements	Flexible policies influence retention and career progression decisions ( Kossek et al., 2011; Kelly et al., 2014).
	**Individual career decisions**	Career choices, negotiation behavior, mobility	Women’s career decisions are shaped by perceived barriers and opportunity structures ( Babcock & Laschever, 2003; Ng et al., 2005; Sullivan & Mainiero, 2008).
		Leadership identity development	Identity formation influences pursuit of leadership roles ( Ely et al., 2011; Lord & Hall, 2005).
**Outcomes**	**Career outcomes**	Leadership representation, promotion rates, wage equity	Gender disparities persist in executive roles and compensation ( Bertrand & Hallock, 2001; Blau & Kahn, 2017; Catalyst, 2023).
		Career advancement and glass ceiling persistence	Despite progress, invisible barriers continue to limit upward mobility ( Cotter et al., 2001; Eagly & Carli, 2007).
	**Organizational outcomes**	Diversity performance, innovation, firm performance	Gender-diverse leadership enhances innovation and financial outcomes ( Post & Byron, 2015; Dezső & Ross, 2012; Hunt et al., 2020).
		Organizational culture transformation	Inclusive practices improve employee engagement and retention ( Shore et al., 2011; Nishii, 2013).
	**Societal outcomes**	Gender equality, economic participation, social inclusion	Increased female leadership contributes to broader societal equity ( World Bank, 2022; UN Women, 2022).
		Sustainable development and governance outcomes	Gender-inclusive leadership aligns with SDG 5 and governance improvements ( UNDP, 2021; OECD, 2020).

Source: Author’s own

## 5. Conclusion

This study shows that the literature on glass ceiling is an emerging interdisciplinary field and is based on four interrelated topics: structural inequalities; organizational mechanisms; human aspects and empowerment strategies; but divided through institutional, geographical canyons. The theoretical bases are still in classical paradigms, and the connexion of new thoughts about intersectionality, sectorial dynamics and digital transformation is sparse. Research networks have a global bias toward developed economies (USA, UK, India, China, Germany, Spain and Italy) and Western journals creating blind spots about gender-specific career barriers in African, Latin American and developing Asian settings. By combining knowledge domain visualization with thematic integration and proposing an ADO-based conceptual framework, this study offers a comprehensive synthesis of contemporary glass ceiling scholarship. The dominance of management-oriented literature implies glass ceiling is mainly pictured as an organizational issue that needs HR solutions and may therefore underestimate sociological, psychological, and systemic level considerations.

## 6. Future scope

Future research should prioritize six underexplored areas:

(i) Intersectionality and intersectional glass ceilings: Most research treats “women” as homogeneous. Subsequent research should investigate the intersection of race, ethnicity, class, sexuality, disability, immigration status and nationality on gender-specific career barriers. How do intersectional oppressions frame distinct pathways to and through leadership?
(ii) Sectoral-Specific Occupational and Industrial Dynamics: The literature has still not provided detailed comparative sector level studies. Deep dives into gender dynamics within emerging fields (digital tech, renewables, AI) versus established strongholds (finance, military, construction) would elucidate the ways in which sector conditions shape barriers and remedies.
(iii) Digital Transformation and Remote Work: Post-pandemic transitions into hybrid/remote, the growing importance of digital platforms and AI, as well as evolving organizational setups reshape career contextual factors. To what extent do these technologies support or complicate traditional glass ceilings?
(iv) Global South/Non-Western Contexts: The research focus is overwhelmingly biased in favour of North America, Western Europe and advanced Asian economies. Research on women’s careers in Africa, South America, South Asia and Southeast Asia would reveal how colonialism, economic patterns and cultural norms have produced gendered barriers distinct to each place.
(v) Integration of Structural-Cultural-Psychological Models: We suggest that future research expand beyond disciplinary boundaries in developing models to theorize about how structural, organizational, cultural, and individual social/psychological factors together interact with and respond to one another to influence women’s career pathways.
(vi) Longitudinal and Life-Course Perspectives: Most research is cross-sectional. Longitudinal designs following women across career transitions, life stages, and organizational contexts would reveal how barriers accumulate, how critical periods operate, and how interventions have sustained impact.

## 7. Implications

Theoretically, this study is one of the first calling for integrated (i.e., multi-level and longitudinal) frameworks addressing intersectionality and critical perspectives to examine glass ceiling dynamics; and practically by showing that meaningful progress awaits systemic organizational changes instead of attitude or implementer based diversity changes. Transparent processes for promotion and pay need to be embedded in institutions with flexible career structures, inclusive cultures and mentoring and development of leadership ongoing while cohesive legislative, institutional, educational and cultural interventions are designed by policy makers; some of which could involve legislation on pay transparency or support for childcare or gender audits or accountability mechanisms to achieve long-term gender equity.

## 8. Limitations

This study is limited by its reliance on Scopus-indexed, English-language journal articles (2015–2025), potentially biasing findings toward mainstream and developed-country scholarship and excluding gray literature and historical depth. Additionally, disciplinary coverage may be incomplete, and the thematic clusters reflect interpretive synthesis rather than a definitive or deterministic framework.

## Data Availability

This work contains the following Underlying data Repository name: **Zenodo.** Title of project - Beyond the Transparent Barrier, https://doi.org/10.5281/zenodo.19591397 ( Tyagi & Singh, 2026). The project contains the following underlying data:
• Glass Ceiling CSV scopus_export_Jan 21-2026_73fc7287-c9e9-48a8-93b9-0198482e15cd.csv (Raw data) License: Data is available under Creative Commons Attribution 4.0 International. This work does not contain any Extended data.
