# Supplementary material for: Beyond the Transparent Barrier: A Domain Visualization and Integrative Review of Contemporary Research on Gender-Based Professional Stasis
Source: F1000Res. 2026 May 29;15:835. [Version 1] doi: 10.12688/f1000research.180295.1 (PMC13329365; doi:10.12688/f1000research.180295.1)
Supplement: Peer review history

# Reviewer report: Reviewer response for version 1

**DOI:** 10.5256/f1000research.198896.r497561

**Published:** 2026-07-02

**Authors:** Aditya Ranjan

**Affiliations:** 1 ITM University, Gwalior, Madhya Pradesh, India

**SUMMARY OF THE ARTICLE**

 This manuscript offers a PRISMA-guided systematic literature review combined with bibliometric and thematic analysis of research on the glass ceiling and gender-based professional barriers. Drawing on 186 peer-reviewed articles indexed in Scopus over the period 2015–2025, the authors employ bibliographic coupling, co-citation analysis, and keyword co-occurrence mapping using VOSviewer to examine the intellectual structure of the field. The study identifies four major thematic areas: gender inequality in academic and labor markets; organizational mechanisms and career pathways; leadership development and career identity; and diversity initiatives and empowerment strategies. Building on these findings, the authors propose an integrative Antecedents–Decisions–Outcomes (ADO) framework to synthesize multi-level drivers and consequences of the glass ceiling phenomenon.

**Clarity, Accuracy, and Literature Review**

**Answer:** **Yes**

 The manuscript is clearly written overall and presents its arguments in a logical sequence from background to methodology, results, and conceptual synthesis. The topic is timely, relevant, and well contextualized within the broader literature on gender inequality, leadership, and career advancement. The review draws on a substantial body of literature and demonstrates good familiarity with the field’s major concepts and debates.

 At the same time, the manuscript would benefit from more careful language editing. Several sections, particularly in the thematic analysis, contain repetitive phrasing and occasional grammatical awkwardness that detract from readability. The literature review is broad and current, but in some places, it becomes descriptive rather than critically integrative. A tighter synthesis of the most influential conceptual and empirical contributions would strengthen the scholarly contribution.

**Study Design and Technical Soundness**

**Answer:** **Yes**

 The study design is appropriate for the objectives stated. A PRISMA-guided systematic review combined with bibliometric mapping is a sound approach for identifying research clusters, intellectual foundations, and thematic evolution in an established and expanding field. The use of bibliographic coupling, co-citation analysis, and keyword co-occurrence is methodologically suitable for domain visualization. The three-part analytical structure systematic screening, bibliometric analysis, and thematic interpretation is coherent and well aligned with the research aims. The proposal of an ADO-based framework is also a useful attempt to integrate findings across structural, organizational, and individual levels. Overall, the study design is technically robust and appropriate to the nature of the inquiry.

**Methods and Replicability**

**Answer:** **Yes**

 The methods are described with reasonable clarity and appear sufficient for replication at a basic level. The authors specify the database used, the time frame, the language restriction, the article type, and the key terms employed in the search. The use of PRISMA screening and the application of VOSviewer are both clearly stated. The availability of underlying data through Zenodo is a notable strength and supports transparency. However, the methodological section would benefit from greater detail regarding the exact search syntax, the number of records excluded at each screening stage, and the specific VOSviewer settings used to generate the network maps. Providing these details would improve reproducibility and methodological rigor.

**Statistical Analysis (if applicable)**

**Answer:** **Not applicable**

 The manuscript does not involve conventional statistical testing. The bibliometric mapping and thematic synthesis are appropriate for the review’s objectives and do not require inferential statistical analysis. The analytical approach is suitable and consistent with the study design.

**Data Availability and Reproducibility**

**Answer:** **Yes**

 The manuscript includes a clear data availability statement and indicates that the dataset is deposited in Zenodo. This is a significant strength. The availability of the Scopus export file and the associated license improves transparency and enables independent verification or extension of the analysis. The study thus demonstrates a commendable commitment to open research practices.

**Validity of Conclusions**

**Answer:** **Yes**

 The conclusions are generally supported by the results. The thematic clusters identified in the manuscript are consistent with the bibliometric evidence, and the proposed ADO framework is a reasonable conceptual synthesis of the field. The authors also identify several important gaps, including the limited attention to intersectionality, sector-specific variation, and underrepresentation of Global South contexts. The future research agenda is logical and relevant, and it follows from the patterns observed in the review. While some claims could be phrased more cautiously given the interpretive nature of bibliometric synthesis, the conclusions are largely valid and appropriately grounded in the data.

**Final Assessment**

 This manuscript makes a useful and timely contribution to the literature on glass ceiling research. Its principal strengths are the combination of systematic review and bibliometric methods, the transparent data availability, and the attempt to develop a higher-level conceptual framework. The main limitations concern language quality, some repetition in the narrative, and the need for fuller methodological reporting. Overall, the manuscript is suitable for indexing after minor revision, with attention to language polishing, tightening of the synthesis, and additional methodological clarification.

Is the work clearly and accurately presented and does it cite the current literature?

Yes

If applicable, is the statistical analysis and its interpretation appropriate?

Not applicable

Are all the source data underlying the results available to ensure full reproducibility?

Yes

Is the study design appropriate and is the work technically sound?

Yes

Are the conclusions drawn adequately supported by the results?

Yes

Are sufficient details of methods and analysis provided to allow replication by others?

Yes

Reviewer Expertise:

Tourism and Hospitality

I confirm that I have read this submission and believe that I have an appropriate level of expertise to confirm that it is of an acceptable scientific standard.

# Reviewer report: Reviewer response for version 1

**DOI:** 10.5256/f1000research.198896.r490120

**Published:** 2026-06-12

**Authors:** Mahesh Uniyal

**Affiliations:** 1 Assam Skill University, Mangaldoi, Assam, India

**SUMMARY OF THE ARTICLE**

 This manuscript presents a PRISMA-guided systematic bibliometric and thematic review examining the intellectual landscape of glass ceiling research on gender-based professional barriers. The authors analyzed 186 peer-reviewed publications from the Scopus database spanning 2015-2025, employing PRISMA screening alongside VOSviewer software for bibliometric analysis. The study addresses two primary research objectives: (1) Identify intellectual structures through bibliographic coupling, co-citation analysis, and keyword co-occurrence; (2) Highlight underexplored areas and propose future research directions. The analysis reveals dominant contributions from USA, UK, India, China with four thematic clusters: Gender Inequality in Academic/Labor Markets, Organizational Mechanisms & Career Pathways, Leadership Development & Career Identity, and Diversity Initiatives & Empowerment Strategies. The manuscript contributes an Antecedents-Decisions-Outcomes (ADO) conceptual framework synthesizing multi-level glass ceiling drivers.

1. **Clarity, Accuracy, and Literature Review**

**Answer:** **☐** **Yes**

 The manuscript is clearly and accurately presented. The authors have structured the paper logically from introduction through bibliometric findings, thematic analysis, to conceptual framework. Language is precise with key concepts (glass ceiling, ADO framework) well explained. The literature review is comprehensive, citing 186 relevant studies across management, sociology, and gender studies from 2015-2025. References are current and highlight field evolution from organizational barriers to intersectional approaches. However, repetitive citations in thematic sections slightly reduce readability.

1. **Study Design and Technical Soundness**

**Answer:** **☐** **Yes**

 The study design is appropriate for objectives. PRISMA systematic review combined with bibliometric analysis (bibliographic coupling, co-citation, keyword co-occurrence) using VOSviewer is technically robust and well-suited for intellectual mapping. Three-phase approach (PRISMA→bibliometric→thematic synthesis) comprehensively addresses research questions. ADO framework development logically follows findings. Methodology ensures reliability across global collaboration patterns and thematic evolution.

1. **Methods and Replicability**

**Answer:** **☐** **Yes**

 Authors provide sufficient methodological details for replication. PRISMA screening from Scopus (2015-2025, English peer-reviewed, glass ceiling keywords) clearly explained with inclusion/exclusion criteria. VOSviewer analyses (bibliographic coupling of countries/documents/sources, co-citation of authors/sources, keyword co-occurrence) well-described. Search strategy, database, timeframe, and tools enable replication. Zenodo dataset availability confirms transparency.

1. **Statistical Analysis (if applicable)**

**Answer:** **☐** **Not applicable**

 Study employs bibliometric network analysis via VOSviewer rather than traditional statistics. Visual cluster mapping and co-occurrence networks appropriate for research design—no hypothesis testing required. Methodologically sound for domain visualization.

1. **Data Availability and Reproducibility**

**Answer:** **☐** **Yes**

 Underlying data fully available via Zenodo repository containing Scopus export CSV (scopus_export_Jan 21-2026). Creative Commons Attribution 4.0 license enables verification and extension. Detailed data retrieval process ensures independent reproduction of bibliometric analyses.

1. **Validity of Conclusions**

**Answer:** **☐** **Yes**

 Conclusions adequately supported by results. Four thematic clusters directly derived from keyword co-occurrence (Figure 7). ADO framework synthesizes antecedents (structural biases), decisions (organizational/individual), outcomes (leadership gaps) across bibliometric findings. Identified gaps (Africa/Latin America, intersectionality) reflect country/source analyses. Six future directions logically address disciplinary fragmentation.

Is the work clearly and accurately presented and does it cite the current literature?

Yes

If applicable, is the statistical analysis and its interpretation appropriate?

Yes

Are all the source data underlying the results available to ensure full reproducibility?

Yes

Is the study design appropriate and is the work technically sound?

Yes

Are the conclusions drawn adequately supported by the results?

Yes

Are sufficient details of methods and analysis provided to allow replication by others?

Yes

Reviewer Expertise:

Medical & Wellness Tourism, Adventure Tourism, Sustainable Tourism, CBT, Hospitality

I confirm that I have read this submission and believe that I have an appropriate level of expertise to confirm that it is of an acceptable scientific standard.
